# Hypoxia-induced responses by endothelial colony-forming cells are modulated by placental growth factor

**DOI:** 10.1186/s13287-016-0430-0

**Published:** 2016-11-29

**Authors:** Michelle B. Hookham, Imran H. A. Ali, Christina L. O’Neill, Emer Hackett, Melanie H. Lambe, Tina Schmidt, Reinhold J. Medina, Sara Chamney, Bharathi Rao, Eibhlin McLoone, David Sweet, Alan W. Stitt, Derek P. Brazil

**Affiliations:** 1 Centre for Experimental Medicine, Queen’s University Belfast, 97 Lisburn Road, Belfast, BT9 7BL UK; 2 Eye & Ear Clinic, Royal Victoria Hospital, Grosvenor Road, Belfast, BT12 6BA UK; 3 Regional Neonatal Unit, Royal Maternity Hospital, Grosvenor Road, Belfast, BT12 6BA UK

**Keywords:** Hypoxia, Endothelial progenitor cells, Endothelial colony-forming cells, Placental growth factor, Senescence, Migration

## Abstract

**Background:**

Endothelial colony-forming cells (ECFCs), also termed late outgrowth endothelial cells, are a well-defined circulating endothelial progenitor cell type with an established role in vascular repair. ECFCs have clear potential for cell therapy to treat ischaemic disease, although the precise mechanism(s) underlying their response to hypoxia remains ill-defined.

**Methods:**

In this study, we isolated ECFCs from umbilical cord blood and cultured them on collagen. We defined the response of ECFCs to 1% O_2_ exposure at acute and chronic time points.

**Results:**

In response to low oxygen, changes in ECFC cell shape, proliferation, size and cytoskeleton phenotype were detected. An increase in the number of senescent ECFCs also occurred as a result of long-term culture in 1% O_2_. Low oxygen exposure altered ECFC migration and tube formation in Matrigel®. Increases in angiogenic factors secreted from ECFCs exposed to hypoxia were also detected, in particular, after treatment with placental growth factor (PlGF). Exposure of cells to agents that stabilise hypoxia-inducible factors such as dimethyloxalylglycine (DMOG) also increased PlGF levels. Conditioned medium from both hypoxia-treated and DMOG-treated cells inhibited ECFC tube formation. This effect was reversed by the addition of PlGF neutralising antibody to the conditioned medium, confirming the direct role of PlGF in this effect.

**Conclusions:**

This study deepens our understanding of the response of ECFCs to hypoxia and also identifies a novel and important role for PlGF in regulating the vasculogenic potential of ECFCs.

**Electronic supplementary material:**

The online version of this article (doi:10.1186/s13287-016-0430-0) contains supplementary material, which is available to authorized users.

## Background

Since the first description of endothelial progenitor cells (EPCs) in 1997 as bone marrow-derived vascular precursors with reparative potential [1], there has been considerable interest in their utility for cell-based therapy of ischaemic vasculopathies. “EPC” refers to a broad range of vascular progenitors characterised using diverse cell surface markers and functional endpoints. Endothelial colony-forming cells (ECFCs), also known as late EPCs or outgrowth endothelial cells (OECs), are a subset of circulating EPCs with the ability to differentiate into endothelial cells and mediate vascular repair [2]. When isolated, ECFCs have significantly higher proliferative capacity compared with mature endothelial cells, and they maintain their endothelial progenitor phenotype during long-term ex-vivo expansion [3]. ECFCs are also capable of supporting vascular repair in vivo by integration into pre-existing vasculature, significantly reducing areas of ischaemia in murine models of retinopathy, myocardial infarction and stroke [4].

ECFCs have potential for use as cell-based therapy to treat ischaemic diseases by aiding vascular repair as well as establishing new vasculature [5]. However, the function and number of ECFCs and other EPC subtypes have been reported to be impaired in a range of vascular disease states such as diabetes [6, 7], cardiovascular disease [8], preeclampsia [9] and vascular development in premature infants [10]. The number and function of ECFCs has also been shown to be impaired in low birth weight infants [11], a patient cohort at risk of retinal ischaemia in infancy and cardiovascular disease in adulthood. Accelerated senescence in premature ECFCs as a result of decreased SIRT1 expression was also demonstrated recently [12]. Therefore, it is important to understand what happens when ECFCs enter a hypoxic environment and what molecular cues are required to stimulate ECFC-mediated vascular repair.

Placental growth factor (PlGF) is a member of the vascular endothelial growth factor (VEGF) family that has been demonstrated to promote angiogenesis both independently and synergistically via heterodimer formation with VEGF-A to activate Flt1/VEGF receptor-1 signalling [13]. PlGF also binds to the neuropilin-1/VEGF165 receptor and stimulates angiogenesis [14]. Since its discovery in 1991 [15], PlGF has been associated with a number of disease states [16] including ischaemia [17], cardiovascular disease [18], cancer [19], arthritis [20] and preeclampsia [21]. Recent reports have revealed that there is increased secretion of PlGF from ECFCs isolated from end-stage renal failure patients [22] while in-vitro exposure to PlGF can enhance tubulogenic function in ECFCs [18]. These findings suggest that PlGF may have an important if ill-defined role in normal ECFC vasoreparative function. The signalling and functional responses of ECFCs exposed to PlGF remains to be elucidated, especially in the context of vascular insufficiency and hypoxia encountered by cells as they home to areas of localised tissue damage [23]. In this study, we define the effects of acute and chronic hypoxia on ECFCs in the absence of other confounding factors. In response to low oxygen, we observed changes in ECFC cell size, shape, cytoskeletal structure and proliferative potential. Low oxygen altered ECFC scratch wound repair and in-vitro tube formation in Matrigel®. Of particular interest, we observed increased levels of PlGF secreted from ECFCs exposed to hypoxia and dimethyloxalylglycine (DMOG), a HIF stabilising agent. Conditioned medium from these cells inhibited tube formation in fresh ECFCs. This effect was reversed by neutralising antibodies against PlGF, confirming its central role in this effect. Improved knowledge of how ECFCs respond to low oxygen in vitro will aid understanding of how they may adapt their function to aid vascular repair in ischaemic diseases.

## Methods

### ECFC isolation, in-vitro culture and hypoxia exposure

ECFCs were isolated from umbilical cord blood of full-term neonates. Mononuclear cells (MNCs) were obtained by density gradient fractionation, re-suspended in complete medium (EGM-2; Lonza) supplemented with 10% FBS and seeded on 24-well culture plates pre-coated with rat tail collagen type 1 (BD Biosciences) at a density of 1 × 10^7^ cells/ml. Colonies typically appeared within 2–3 weeks, and ECFC clones were typically cultured for >20 passages. A humidified, temperature-controlled hypoxia chamber (Coy Laboratories) was used to maintain cells in 1% O_2_. Culture media were preconditioned to 1% O_2_ prior to use to ensure instantaneous hypoxia exposure and the temperature was maintained at 37 °C. A Casy cell counter-analyser (Roche) was used for cell counts, viability and cellular size assessment.

### Immunocytochemistry

Cells were fixed in 4% (w/v) paraformaldehyde for 20 min at room temperature. Following PBS washes, cells were permeabilised with 0.1% Triton X-100 for 10 min at RT and then blocked in 1% bovine serum albumin (BSA) for 2 h in PBS before overnight incubation in primary antibody at 4 °C. After washing with PBS, cells were incubated with appropriate secondary antibody for 1 h at RT and imaged under a confocal fluorescence scanning microscope (Nikon). Primary antibodies phalloidin (Invitrogen) and vinculin (Sigma-Aldrich) were used to stain F-actin and focal adhesion complexes respectively. Anti-mouse Alexa Fluor 568 IgG (Invitrogen) was used as secondary antibody. Mounting medium contained the nuclear stain DAPI (Vectashield; Vector Laboratories).

### Flow cytometry

For staining, 5 × 10^5^ ECFCs were used per sample. Cells were filtered using cell strainers with a 35-μm nylon mesh (BD Falcon; BD Biosciences), re-suspended in 100 μl PBS buffer and incubated with respective antibodies for 45 min at 4 °C. Antibodies reactive to CD31 (platelet endothelial cell adhesion molecule (PECAM)), CD45 (protein tyrosine phosphatase receptor type C), CD14 (monocyte differentiation antigen; eBioscience) and CD146 (melanoma cell adhesion molecule; BD Biosciences) were utilised. After staining, cells were washed in PBS and finally resuspended in 1 ml PBS for analysis using an Attune® Acoustic Focusing Flow Cytometer (Invitrogen Life Technologies). At least 20,000 events were acquired for each sample. Respective isotype controls were used to determine accurate settings for data analysis.

### Protein extraction and western blotting

Protein was extracted from ECFCs using ice-cold RIPA containing protease and phosphatase inhibitors as described previously. Twenty micrograms of protein was resolved by electrophoresis through 10% SDS polyacrylamide gel and electro-transferred onto PVDF membrane (Millipore). The membranes were blocked for 1 h at RT with 3% (w/v) non-fat dry milk in Tris-buffered saline with 0.1% Tween-20 (TBS-T). After blocking, the membranes were incubated overnight at 4 °C with primary antibodies reactive to HIF-1α (#610958; BD Biosciences) and HIF-2α (R&D Systems). After three washes with TBS-T, the membranes were incubated for 1 h with their respective HRP-conjugated secondary antibodies (Santa Cruz). Three TBS-T washes were carried out before the protein bands were detected using ECL (Merck Millipore). Membranes were re-probed with β-actin (Cell Signaling Technology) as a housekeeping gene to ensure equal loading.

### Scratch wound repair assay

ECFCs were grown on collagen-coated 24-well plates until confluent. The monolayer was then scratched using a p200 pipette tip to create a wound. After gentle washing with PBS, the growth media was changed to serum-free EGM-2. The scratched area was imaged using ImageJ analysis software, and the wound distance was measured at the beginning (T_0_) and end of the experiment (T_X_). The following formula was used to convert the migrated area of the scratch wound into a percentage:

$$ \%\  of\  wound\  closure = {T}_0\ \hbox{--}\ {T}_X = X\ /\ {T}_0* 100. $$

### Cell viability

ECFC viability was determined using the Cell Counting Kit-8 (CCK-8; Sigma) as per the manufacturer’s instructions. Cells were seeded in 96-well collagen-coated plates (1 × 10^4^ cells/well). Cells were treated for 24 h with 100 ng/ml human recombinant PlGF (R&D Systems) or vehicle (4 mM HCl 0.1% BSA). Absorbance was measured at 450 nm using an Omega plate reader.

### Proteome profiling

A proteome profiler human angiogenesis array (R&D Systems) was used to analyse the secretion of angiogenic cytokines from ECFCs. Cells were maintained in serum-free medium in 21 and 1% O_2_ for 24 h, in accordance with the manufacturer’s instructions. ImageJ image analysis software was used to calculate densitometry and all results were normalised to a positive control. PlGF levels in cell culture supernatant were measured using a PlGF-specific ELISA (R&D Systems) according to the manufacturer’s instructions.

### DMOG treatment

ECFCs were grown in complete media (EGM-2; Lonza) and treated with either dimethylsulfoxide (DMSO, vehicle) or 1 mM DMOG (Sigma-Aldrich) for 24 h. At harvest, media were removed and stored at –80 ^o^C for analysis, after which the cells were washed in ice-cold PBS prior to further analysis by western blot analysis. PlGF levels in cell culture supernatant were measured using a PlGF-specific ELISA (R&D Systems) according to the manufacturer’s instructions.

### Tubulogenesis assay

ECFCs were labelled using a fluorescence membrane labelling kit (PKH; Sigma) or Calcein AM dye (Molecular Probes) according to the manufacturer's protocol. Between 8 × 10^4^ and 1 × 10^5^ labelled ECFCs were re-suspended in basement membrane matrix (Matrigel®; BD Biosciences). Fifty-microlitre aliquots were spotted onto four-well plates. After polymerisation, the spots were covered in endothelial cell growth medium (EGM-2; Lonza) or a 1:1 ratio of EGM-2 and conditioned media. Wells were assessed for the presence of tube-like structures after 24, 48 and 72 h, and were imaged using a confocal scanning microscope (Nikon). For PlGF neutralising experiments, ECFCs (7.5 × 10^5^ cells) were mixed with conditioned medium collected from previously treated ECFCs exposed to vehicle or DMOG (1 mM) and resuspended in basement membrane Matrix (Matrigel®). Next, 50-μl aliquots were spotted onto a 24-well plate and allowed to polymerise. After polymerisation, spots were covered in conditioned medium from vehicle or DMOG-treated cells in the presence of a PlGF neutralising antibody (MAB264; R&D Systems) or an isotype IgG control (Sigma). Matrigel® spots were labelled with Calcein AM dye (Molecular Probes) prior to imaging. Wells were assessed for the presence of tube-like structures after 24 h, and imaged using a confocal scanning microscope (Nikon). The vascular tube area was quantified using NIS Elements software (Nikon).

### Reverse-transcription and real-time PCR

Total RNA was isolated using an RNeasy Mini Kit (Qiagen). cDNA was synthesised from 500 ng RNA using Superscript III (Invitrogen). Semi-quantitative real-time PCR was performed using gene-specific Real Time ready Custom Single assays for ANGPTL4, SLC2A1 and housekeeping genes β-actin and 18S (Roche). Relative quantitative values were obtained using the ∆∆Ct method.

### β-Galactosidase activity

ECFCs were grown on collagen-coated glass coverslips prior to senescence analysis. β-Galactosidase activity was detected using the Senescence β-galactosidase Staining Kit (Cell Signalling Technology) according to the manufacturer’s instructions. The cell monolayer was imaged using a phase contrast microscope (Nikon). The percentage of positively stained cells was quantified using ImageJ image analysis software.

### Statistical analysis

Data were analysed using Student’s two-tailed paired or unpaired *t* test or ANOVA and were plotted as mean ± standard deviation (SD) unless otherwise indicated.

## Results

**Fig. 1:**
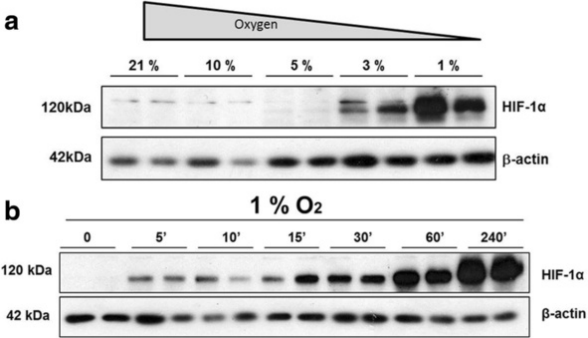
HIF-1α stabilisation in ECFCs occurs rapidly at 1% O_2._ ECFCs were grown in 12% FCS-supplemented EBM-2 medium. **a** ECFCs were cultured at the indicated oxygen tension for 5 h. Protein lysates were probed using HIF-1α (*top panel*), with β-actin included as a loading control (*bottom panel*). **b** ECFCs were cultured in full EBM medium at 1% O_2_ for the indicated time range, starting at 5 min (*5’*) and ending at 240 min (*240’*). Cells were lysed and protein lysates were probed with HIF-1α (*top panel*), with β-actin included as a loading control (*bottom panel*) (*n* = 3). *HIF-1α* hypoxia inducible factor 1 alpha

ECFCs were isolated from umbilical cord blood of normal term infants as described previously [24]. The endothelial character of these cells was confirmed by the expression of PECAM (CD31) and melanoma metastasis-associated surface molecule (MUC18, CD146) (Additional file 1: Figure S1). In addition, isolated ECFCs did not express the leukocyte markers CD45 and the myeloid marker CD14, which confirmed that these cells were not hematopoietic in nature (Additional file 1: Figure S1). To examine the effects of low oxygen on these cells, ECFCs were grown in a range of oxygen tensions from 21% (atmospheric oxygen_,_ 760 mmHg) to 10, 5, 3 and 1% (36 mmHg, Fig. 1a). HIF-1α immunoreactivity was only detected at ≤3% O_2_, with the highest levels of HIF-1α detected at 1% (Fig. 1a). The inhibition of prolyl-hydroxylases and accumulation of cytoplasmic HIF-1α at 1% O_2_ was extremely rapid, and was detected within 5 min of hypoxia exposure (Fig. 1b). A time-dependent increase in HIF-1α accumulation occurred in ECFCs, with the strongest accumulation present after 4 h (240 min) of hypoxia exposure. Based on these data, all subsequent hypoxia exposure experiments were carried out at 1% O_2_ for a minimum of 4–5 h.

**Fig. 2:**
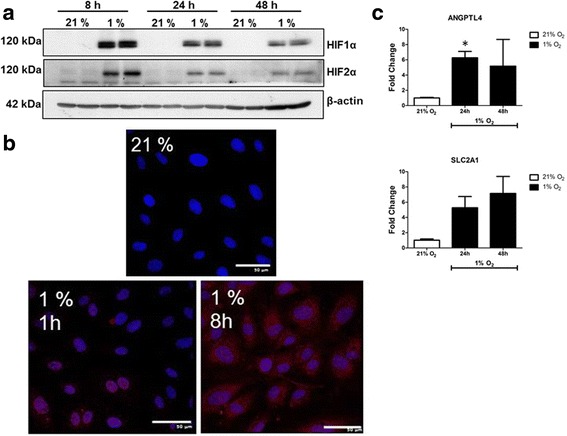
ECFC responses to 1% O_2_. ECFCs were grown in 12% FCS-supplemented EBM-2 medium and maintained in either 21 or 1% O_2_ for the indicated times. **a** Protein lysates isolated from time-matched ECFCs grown under normal conditions (21% O_2_) or hypoxic conditions (1% O_2_) were probed with HIF-1α (*top panel*) or HIF-2α, with β-actin included as a loading control (*bottom panel*). **b**. ECFCs cultured at 21 or 1% for 5 h were permeabilised and incubated with antibodies reactive to HIF-α (*red*) and DAPI to stain the nuclei (*blue*). *Scale bar*: 50 μM. **c** Real-time PCR was carried out on cDNA generated from ECFCs incubated in 1% O_2_ for the indicated times. Levels of HIF-1 responsive genes ANGPTL4 and SLC2A1 were measured, and normalised to the mean of two housekeeping genes (18S and β-actin). The ∆∆Ct subtractive method was used to compare between treatments, with 21% O_2_ as the calibrator. Fold-changes compared with 21% O_2_ were calculated. Data plotted as mean fold-change ± SD. *p < 0.05 (*n* = 5). *HIF-1α* hypoxia inducible factor 1 alpha (Colour figure online)

Accumulation of both HIF-1α and HIF-2α isoforms were observed in ECFCs grown in 1% O_2_ for 8, 24 and 48 h (Fig. 2a). In comparison with ECFCs grown in 21% O_2_ (160 mmHg), cells grown in 1% O_2_ demonstrated a marked increase in HIF-1α immunoreactivity in the cytosol, with punctate staining in the nuclei evident at the 5 h time point (Fig. 2b). This suggested that the HIF-1α subunit was translocating to the nucleus as part of the typical transcriptional response to hypoxia [25]. This was further corroborated by the increased expression of HIF-1α regulated transcripts ANGPTL4 and SLC2A1 (also known as GLUT1) in ECFCs grown in 1% O_2_ (Fig. 2c). Thus, exposure to low oxygen concentrations induces a typical “hypoxia” response in terms of HIF-1α transcription factor accumulation and gene transcription in ECFCs.

**Fig. 3:**
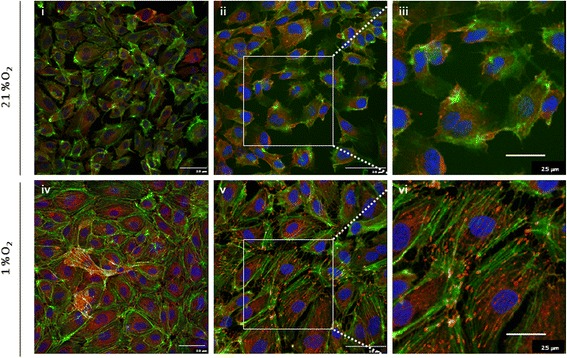
Hypoxia induces actin rearrangement and stress fibre formation in ECFCs. The ECFCs were grown in 12% FCS-supplemented EBM-2 medium and maintained in either 21 or 1% O_2_ for 24 h. ECFCs were stained with phallodin to identify filamentous actin (F-actin, *green*), vincullin (*red*) to identify focal adhesions and DAPI to stain the nuclei (*blue*). *i, iv* 40 × magnification, *ii, v* 60 × magnification. *i, ii, iv, v Scale bars*: 50 μm size marker. *ii, v* Magnified area depicted in *iii* and *vi*. Actin stress fibres (*green*) and focal adhesions (*red*) are evident in a single cell grown in 1% O_2_ (*iii, vi*). *iii, vi Scale bar*: 25 μm size marker (*n* = 3)

**Fig. 4:**
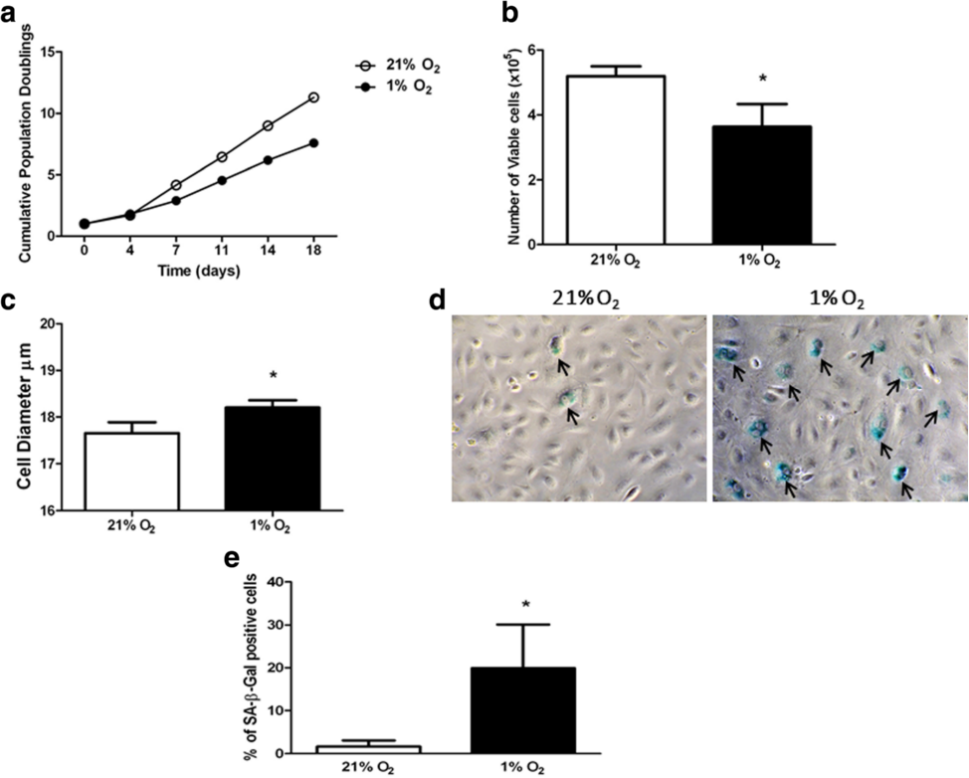
Long-term exposure of ECFCs to hypoxia induces partial senescence. **a** ECFCs cultured at 21% O_2_ (*open circles*) or 1% O_2_ (*filled circles*) for the indicated times. At each passage, cells were counted using a Casy cell counter. **b** Number of viable cells after 10-day culture in 21% O_2_ (*empty bars*) versus 1% O_2_ (*filled bars*). Cell numbers plotted as mean number of viable cells ± SD. *p < 0.05. **c** Diameter of cells in culture after 10-day maintenance in 21% O_2_ (*empty bars*) versus 1% O_2_ (*filled bars*). Data plotted as mean cell diameter (μm) ± SD. *p < 0.05. **d** ECFCs in culture were subjected to SA-β-galactosidase staining after 10-day hypoxia exposure (1% O_2_) together with time-matched normoxia controls (21% O_2_). Representative images using bright-field microscopy, magnification 20 ×. **e** Number of SA-β-galactosidase-positive ECFCs in 21% (*open bars*) and 1% O_2_ (*filled bars*) counted and expressed as a percentage of total cells. Data plotted as mean percentage ± SD. *p < 0.05 (*n* = 3)

**Fig. 5:**
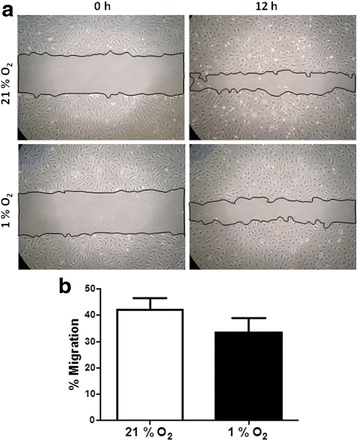
Hypoxia delays scratch wound repair and tube formation in ECFCs. The ECFCs were grown in 21 and 1% O_2_. Cells were photographed at the time of scratch and at 12 h post wounding. **a** Representative images of scratch wound at time zero (*0 h*) and 12 hours (*12 h*). *Bars* indicate the boundaries of the scratch measured at each time point. **b** Percentage migration 12-h post scratch. Each condition was compared separately and the percentage migration calculated for 21% O_2_ (*empty bars*) or 1% O_2_ (*filled bars*). Data plotted as mean ± SEM of three independent experiments (*n* = 4)

ECFC exposure to hypoxia induced the formation of actin stress fibres, with cells displaying a more organised, linear arrangement of F-actin (Fig. 3ii, v). Vincullin staining also revealed the appearance of focal adhesions in ECFCs grown in 1% O_2_, at the terminal ends of the striated F-actin fibres (Fig. 3vi). These data suggest that low oxygen concentrations triggered cytoskeletal changes in ECFCs suggestive of cellular stress and an adaption to the hypoxic environment by strengthening their attachment to the surface of the culture flasks. To further explore the effect of low oxygen on ECFC phenotype, cells were maintained in 1% O_2_ for up to 18 days. Chronic hypoxia reduced ECFC cell doubling and viability (Fig. 4a, b). Consistent with the cytoskeletal changes seen after 48 h, ECFC cell size increased after culture in 1% O_2_ for 10 days (Fig. 4c). The number of β-galactosidase-positive ECFCs increased significantly after long-term culture in 1% O_2_, suggestive of a senescence-like phenotype in a proportion of these cells (Fig. 4d, e). Together, these data suggest that hypoxia induces changes in ECFC phenotype suggestive of a more adhesive, less mitotic, senescent-like phenotype. To test this hypothesis, scratch wound assays were carried out at 21 and 1% O_2_. Exposure of ECFCs to 1% O_2_ decreased the wound closure rate compared with cells grown in 21% O_2_, although this difference was not significant (Fig. 5a, b). Thus, changes in ECFC cytoskeleton are consistent with an increased adhesive capacity linked to a reduced migratory potential in ECFCs.

**Fig. 6:**
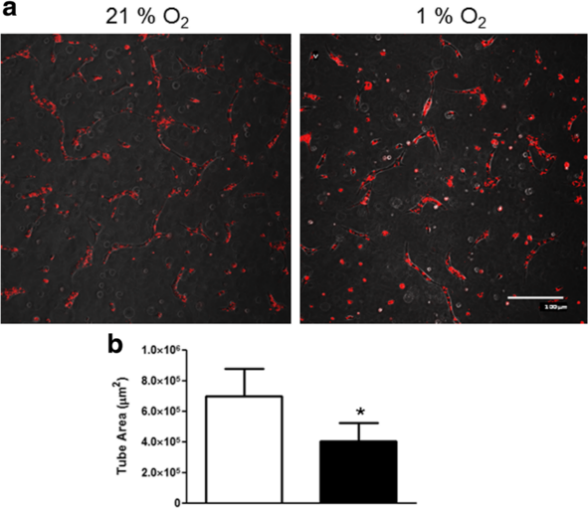
Hypoxia exposure decreases ECFC angiogenesis in vitro. **a** ECFCs were stained with PKH membrane dye and grown in Matrigel®, and the formation of tube-like structures was assessed after 48 h. Representative images for 21 and 1% O_2_ are shown. *Scale bar*: 100 μm. **b** Total tube area (μm^2^) was quantified using NIS Elements software (Nikon) for ECFCs grown in 21% O_2_ (*empty bars*) or 1% O_2_ (*filled bars*). Data plotted as mean ± SD. *p < 0.05. *Scale bars*: 100 μm (*n* = 3)

ECFCs are believed to be recruited from a defined niche into the systemic circulation, after which they home to an area of tissue ischaemia/hypoxia where they stimulate vascular repair and/or new blood vessel formation [26]. In the in-vitro setting, tubulogenesis in Matrigel® is a robust indicator of vasculogenic function of ECFCs growing under normal atmospheric oxygen. Incubation of ECFCs under normal oxygen conditions triggered tube formation that was evident at 48 h (Fig. 6a, b). In contrast, tube area was significantly lower in ECFCs cultured in 1% O_2_. These data suggest that hypoxia decreases ECFC-mediated tube formation in vitro.

**Fig. 7:**
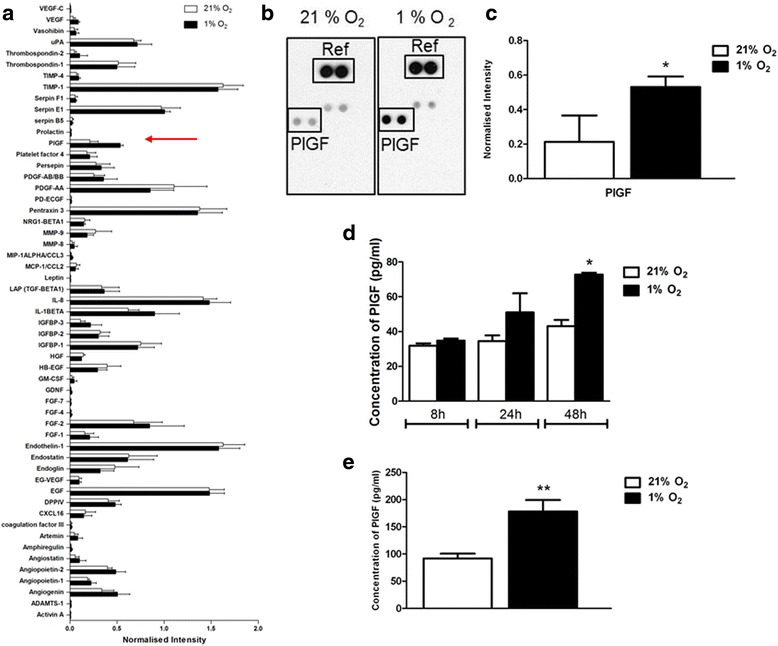
Hypoxia increases secretion of PlGF in ECFCs **a** Conditioned medium from ECFCs grown for 48 h at 21% O_2_ (*empty bars*) or 1% O_2_ (*filled bars*) was incubated on a human angiogenesis Proteome Profiler array to identify soluble factors secreted. *Red arrow*, position of PlGF on the graph. **b**, **c** Signal intensity measured using ImageJ software, and a positive control (*Ref*) was used to normalise the intensity of PlGF between conditions. **d** Conditioned medium from ECFCs grown in 21 and 1% O_2_ was probed using a PlGF-specific ELISA. PlGF concentrations were extrapolated from a standard curve and plotted as mean ± SD. *p < 0.05. **e** ECFCs were grown in 1% O_2_ for 11 days. Conditioned medium was harvested and PlGF levels were measured by ELISA. Data plotted as mean ± SD. **p < 0.01 (*n* = 3). *PlGF* placental growth factor

ECFCs are known to secrete a range of growth factors and cytokines which have autocrine stimulatory function [27], although it is unknown how exposure to hypoxia influences this secretome. A profile of secreted angiogenic factors from the conditioned medium of ECFCs grown in 21 and 1% O_2_ for 24 h was identified using a Proteome Profiler (R&D Systems). When compared with ECFCs growing in normal atmospheric oxygen condition, hypoxia-exposed cells secreted higher levels of VEGF and PlGF (Fig. 7a–c). Consistently, levels of PlGF were significantly increased at 48 h when the conditioned medium of ECFCs grown in 1% versus 21% O_2_ was analysed by ELISA (Fig. 7d). Importantly, increased levels of PlGF were maintained in ECFCs exposed to 1% O_2_ for 11 days, suggesting that PlGF secretion is not only a short-term response to low oxygen, but is also maintained during chronic exposure to hypoxia, which is suggestive of an adaptive response of these cells to a hypoxic environment (Fig. 7e).

**Fig. 8:**
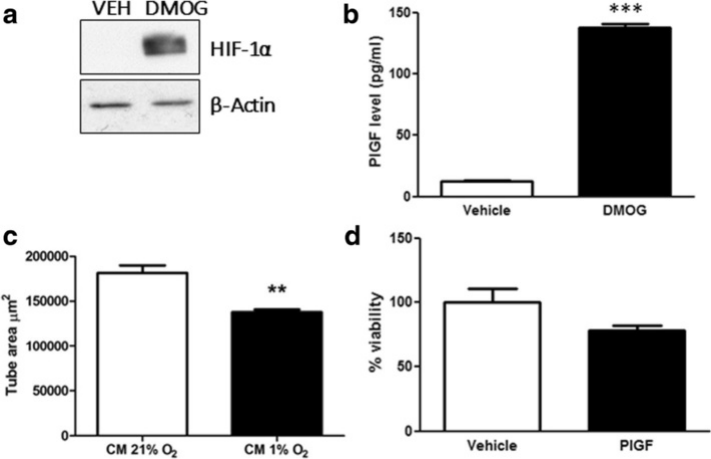
HIF-1α stabilisation induces PlGF expression and alters ECFC tube formation and viability. **a** ECFCs were grown in Matrigel® and exposed to vehicle (DMSO, *VEH*) or DMOG (1 mM) for 24 h. Protein lysates were probed for HIF1α (*upper panel*) or β-actin (*lower panel*). **b** PlGF concentration was measured by ELISA in conditioned medium from ECFCs treated with vehicle (DMSO) or DMOG (1 mM) for 24 h. **c** Conditioned medium (*CM*)from ECFCs treated with vehicle (DMSO) or DMOG (1 mM) was used to treat ECFCs grown in Matrigel® and stained with PKH for 24 h. Total tube area (μm^2^) quantified using NIS Elements software. **d** ECFCs were treated with conditioned medium from vehicle (DMSO) or DMOG (1 mM)-treated cells for 24 h. Cell viability measured using a CCK-8 cell viability assay (Sigma), and plotted as mean % viability ± SD (*n* = 4). *DMOG* dimethyloxalylglycine, *PlGF* placental growth factor. Data plotted as Mean ± SD **p < 0.01; ***p < 0.001

To establish whether this upregulation of PlGF occurred as a result of hypoxia-induced stabilisation of HIF-1α and other transcription factors, ECFCs were treated with dimethyloxalylglycine (DMOG), a cell-permeable inhibitor of prolyl hydroxylases (PHDs) that decreases proteosomal-mediated destruction of HIF-1α. Stabilisation of HIF-1α was observed in ECFCs grown in 21% O_2_ treated with DMOG for 24 h (Fig. 8a). Conditioned medium from DMOG-treated ECFCs also contained higher levels of PlGF, with a higher fold-change than that observed in ECFCs incubated in 1% O_2_ versus 21% O_2_ (Fig. 8b). Consistent with previous data (Figs. 4b and 6), conditioned medium from DMOG-treated ECFCs decreased tube formation and cell viability in ECFCs, mimicking the effect seen in 1% O_2_ (Fig. 8c, d).

**Fig. 9:**
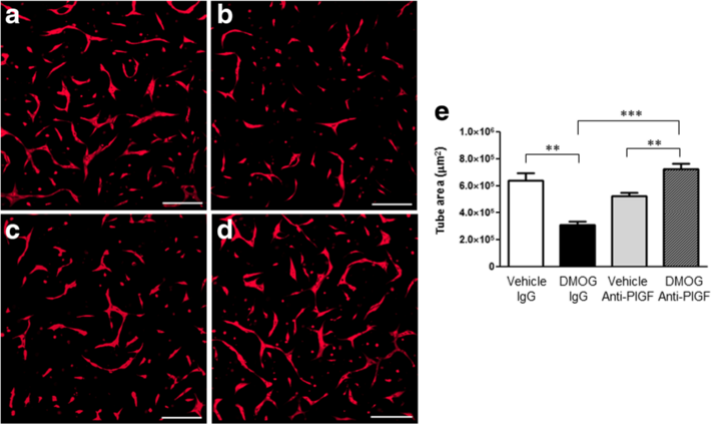
Anti-PlGF neutralising antibody blocks DMOG-induced inhibition of ECFC tube formation. ECFCs were stained with calcein (Invitrogen) and grown on Matrigel®. Cells were treated with conditioned medium harvested from ECFCs treated with **a** DMSO (vehicle) plus isotype control IgG, **b** DMOG plus isotype control IgG, **c** DMSO plus anti-PlGF neutralising antibody or **d** DMOG plus anti-PlGF neutralising antibody. **e** At 48 h, total tube area (μm^2^) was quantified using NIS Elements software (Nikon). Data plotted as mean ± SD and are representative of three independent experiments. **p < 0.01; ***p < 0.001. *Scale bars*: 200 μm (*n* = 3). *DMOG* dimethyloxalylglycine, *PlGF* placental growth factor

To test whether PlGF in the conditioned medium from ECFCs was mediating this inhibition of tube formation, a neutralising PlGF antibody was used to inhibit PlGF in these experiments. Exposure of ECFCs to conditioned medium from DMOG-treated cells inhibited tube formation, as seen previously (Fig. 9, a versus b). Significantly, incubation of conditioned medium from DMOG-treated ECFCs with anti-PlGF neutralising antibody reversed this inhibitory effect and returned tube formation to control levels (Fig. 9, c versus d, e). The inclusion of an isotype antibody control in these experiments suggests that this effect is specifically due to the binding and inhibition of PlGF by the neutralising antibody (Fig. 9c).

## Discussion

ECFCs remain a promising candidate for cell therapy to treat tissue ischaemia. However, robust characterisation of ECFC cell behaviour in the target tissues and disease-related microenvironments is required as these cells move towards therapy. In this study, we have defined the response of ECFCs to hypoxia exposure, one of the main factors associated with vascular insufficiency and tissue ischaemia. The HIF transcription factors are considered master regulators of hypoxia, having an established role in the cellular response to oxygen deprivation, and are of critical importance in hypoxia-driven angiogenesis by controlling the expression of many genes required for the process [28]. In response to a graded decrease in O_2_, HIF-1α stabilisation was detected in ECFCs at the lower O_2_ levels of 3 and 1% with no HIF stabilisation detectable at the higher oxygen concentrations of 5 and 10% (Fig. 1). Similarly, in a study investigating the effect of low oxygen levels on ECFCs and MSCs, Hofmann et al. [29] reported that while MSCs and fibroblasts are very sensitive to lowering oxygen concentration HIF-1α stabilisation only occurred at 1% O_2_ levels. Accumulation of HIF-1α occurred within 5 min in these cells (Fig. 1b). These findings are important because the physiological oxygen tension varies widely between different cell types and tissues. For example, alveoli have a physiological oxygen concentration of approximately 14.5% (approximately 110 mmHg), while the brain has a much lower physiological oxygen percentage in the region of 4.4% (34 mmHg) [30]. In fact, culturing ECFCs in ambient 21% O_2_ may actually elevate the physiological oxygen tension above baseline, where key stem cell niches such as the bone marrow would normally experience a much lower physiological oxygen tension of around 6.4% (49 mmHg) [30]. Other groups have found that culturing induced pluripotent stem cells (iPS) and mesenchymal stem cells (MSCs) at low oxygen concentrations provides enhanced functional outcomes [31, 32]. While not within the remit of this study, future studies will probably define the optimum oxygen concentration for ex-vivo ECFC culture. Maintenance in an oxygen concentration likely to be experienced in vivo may improve the therapeutic potential of these cells for clinical use. For this study, we chose 1% O_2_ based on robust stabilisation of HIF-1α and HIF2α, along with HIF-1α nuclear translocation and hypoxia-dependent gene expression changes that were detected under these conditions (Figs. 1 and 2).

ECFCs cultured in 1% O_2_ for 24–72 h displayed changes in their actin cytoskeleton (Fig. 3), as well as cell doubling, increased cell size and staining for senescence markers (Fig. 4). Increased filamentous actin, along with the appearance of focal adhesions at the end of actin stress fibres, suggested that hypoxia was inducing enhanced ECFC adherence to the cell culture dish (Fig. 3). One interpretation of these data is that when ECFCs are recruited to the ischaemic/hypoxic tissue, they need to attach to the extracellular matrix in close proximity to either damaged blood vessels or avascular regions. These data were supported by reduced ECFC scratch wound repair in 1% O_2_ versus 21% O_2_ (Fig. 5). Reductions in ECFC chemotaxis/migration in response to low oxygen have been reported by both the Yoder and Strunk groups [29, 33], while the Yoder group also detected an increase in hypoxia-mediated tube formation in ECFCs, albeit at an earlier time point than our study (6 h versus 72 h, Fig. 4d [33]). Hofmann et al. [29] also report a decrease in tube formation in ECFCs when exposed to 1% O_2_. In vascular smooth muscle cells, HIF-1α expression decreased cell migration and adherence to the ECM, with no evident changes in vincullin or focal adhesion kinase (FAK) localisation [34]. Hypoxia increased MSC migration, probably via VEGF-mediated phosphorylation of FAK and activation of MAPK and eNOS signalling [35]. Hypoxia increased αvβ3 integrin cell surface expression and cell adhesion in trophoblast stem cells [36]. Together with our results, these data suggest that there may be cell-type specific responses to low oxygen, with ECFCs demonstrating increased adhesiveness and decreased proliferation and migration, perhaps to ensure their effective engraftment to the ischaemic tissue in vivo. Consistently, we have identified that exposure of ECFCs to hypoxia decreases the expression of CD34, a marker of “stemness”, which suggests that ECFCs in the hypoxic niche become more endothelial-like to perhaps improve their ability to repair damaged endothelium (data not shown).

One of the key findings of this study was the increased secretion of PlGF by ECFCs (Fig. 6). Despite the robust increase in PlGF protein expression in 1% O_2_ or in response to DMOG, an inhibitor of HIF prolyl hydroxylases [37], previous analysis of the PlGF promoter and enhancer regions did not identify any the putative binding sites for HIFs (hypoxia response elements (HREs)) [38–40]. Consistent with data from Xiang et al. [41], we did not detect any increase in PlGF RNA in hypoxia-exposed ECFCs. In contrast, overexpression of HIF-1α has been reported by others to augment the expression of PlGF in both endothelial cells [42–44] and primary cardiac and vascular cells [45], indicating that HIFs may well have a role to play in PlGF expression. A recent report identified novel HIF-1α HREs in intron 2 of the PlGF gene, and highlighted chromatin remodelling via histone H3 and H4 acetylation as a key element in HIF1α-mediated PlGF upregulation [44, 46]. Other transcription factors such as NFκB and CREB are also stabilised under low oxygen conditions, and may play a role in PlGF upregulation [41, 42, 45, 46]. PlGF has been reported to enhance angiogenic signalling by acting synergistically with VEGF to increase signalling via the receptor VEGFR2 [47, 48] while also maintaining the ability to induce angiogenic signalling independent of VEGF [49]. Others have identified that PlGF may increase blood vessel formation via monocyte recruitment and paracrine signalling to endothelial cells [41, 44]. Increased PlGF secretion from ECFCs coincided with a delay in in-vitro tube formation in Matrigel® (Fig. 8b, c). Neutralising antibodies against PlGF rescue ECFC tube formation in Matrigel® (Fig. 9). Thus, hypoxia/HIF-mediated increases in PlGF secretion from ECFCs may inhibit tube formation and vasculogenesis.

Our data suggest that, in vitro, in the absence of VEGF, PlGF may not be driving ECFC tube formation, and heterodimers of PlGF and VEGF may be the active moieties. This is somewhat surprising, given the previously described pro-angiogenic activity of PlGF [50]. It remains to be determined whether PlGF released from ECFCs in the hypoxic niche would have both an autocrine and a paracrine function, and what the relative balance of these functions might be. We can also speculate that PlGF from chronically hypoxia-exposed ECFCs may function to attract monocytes, pericytes or mural cells to stabilise the vascular niche. PlGF may also be secreted from the cells of the tissue parenchyma in ischaemic tissue. Alternatively, a paracrine source of VEGF or other factor from cells such as pericytes or endothelial cells may be required to trigger new blood vessel formation. ECFCs have been demonstrated to act as paracrine mediators, modulating MSC regeneration potential prior to the establishment of neovascularisation and blood perfusion, ultimately enabling extensive engraftment and long-term differentiation of transplanted MSCs [51]. Recent work by Xiang et al. [41, 44] supports the idea of a paracrine role for PlGF secreted by endothelial cells in vascular repair.

## Conclusions

This study deepens our understanding of the response of ECFCs to hypoxia and also addresses important questions regarding the role of PlGF and chronic hypoxia in influencing the neovascular potential of ECFCs. Further studies will be required to determine whether ex-vivo pre-conditioning of ECFCs, by either hypoxia or PlGF, or manipulation of PlGF expression in vivo may be useful tools to augment ECFC neovascularisation for the future treatment of ischaemic disease.

## Additional file

Additional file 1: Figure S1. showing characterisation of ECFC identity using flow cytometry. ECFCs were grown in complete EBM2 medium at 21% O_2_. Cells were trypsinised and stained with antibodies against endothelial markers CD31 and CD146 (*green*) and hematopoietic markers CD45 and CD14 (*red*). Respective isotype controls are shown in black and % positivity is shown in the *bottom right-hand corner*. Cell number plotted on *x* axis and fluorescence intensity plotted on *Y* axis. Data are representative of experiments carried out on at least seven ECFC clones (*n* = 7). (TIF 73 kb)

## Abbreviations

ANGPTL4: Angiopoietin-like 4
CREB: cAMP response element-binding protein
DMOG: Dimethyloxalylglycine
ECFC: Endothelial colony-forming cell
eNOS: Endothelial nitric oxide synthase
EPC: Endothelial progenitor cell
FAK: Focal adhesion kinase
GLUT1: Glucose transporter 1
HIF-1α: Hypoxia inducible factor 1 alpha
HIF-2α: Hypoxia inducible factor 2 alpha
MAPK: Mitogen-activated protein kinase
MUC18: Melanoma metastasis-associated surface molecule
NFκB: Nuclear factor kappa-light-chain-enhancer of activated B cells
OEC: Outgrowth endothelial cell
PECAM: Platelet endothelial cell adhesion molecule
PlGF: Placental growth factor
SLC2A1: Solute carrier family 2, facilitated glucose transporter member 1
VEGF: Vascular endothelial growth factor
VEGFR2: Vascular endothelial growth factor receptor 2
