# Supplementary figures and images for: Hypoxia-induced responses by endothelial colony-forming cells are modulated by placental growth factor
Source: Stem Cell Res Ther. 2016 Nov 29;7:173. doi: 10.1186/s13287-016-0430-0 (PMC5129608; doi:10.1186/s13287-016-0430-0)

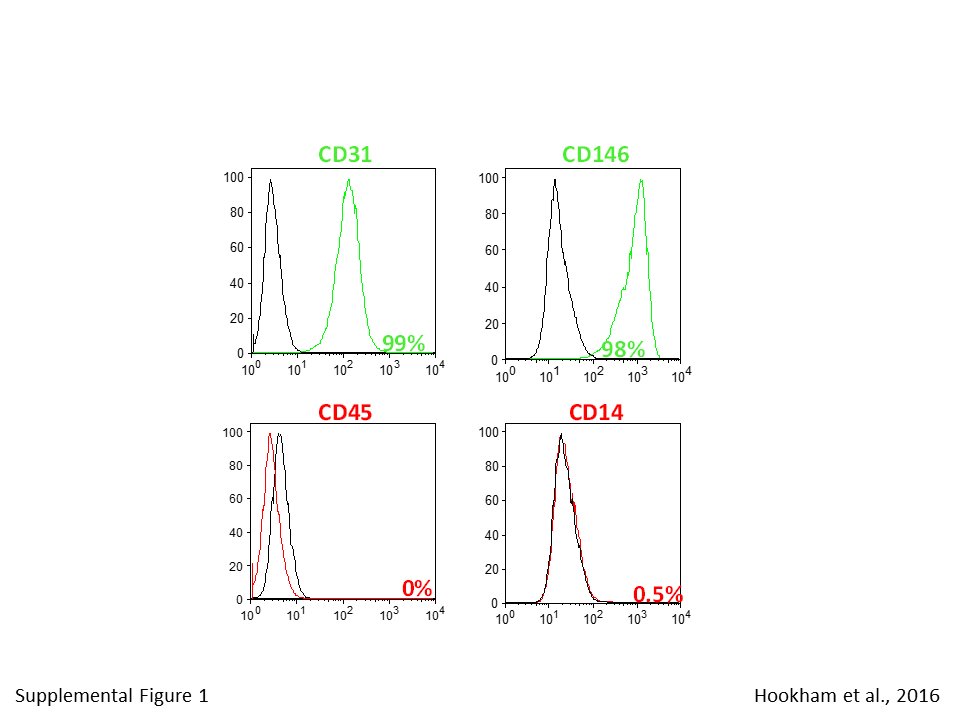

Supplement: Additional file 1: Figure S1. — showing characterisation of ECFC identity using flow cytometry. ECFCs were grown in complete EBM2 medium at 21% O2. Cells were trypsinised and stained with antibodies against endothelial markers CD31 and CD146 (green) and hematopoietic markers CD45 and CD14 (red). Respective isotype controls are shown in black and % positivity is shown in the bottom right-hand corner. Cell number plotted on x axis and fluorescence intensity plotted on Y axis. Data are representative of experiments carried out on at least seven ECFC clones (n = 7). (TIF 73 kb) [file 13287_2016_430_MOESM1_ESM.tif]
